# Corticosteroid-induced exacerbation of cryptic miliary tuberculosis to acute respiratory distress syndrome: A case report

**DOI:** 10.1097/MD.0000000000023204

**Published:** 2020-11-13

**Authors:** Minji Song, Sung Jin Kim, Jin Young Yoo

**Affiliations:** a Department of Radiology, Chungbuk National University Hospital; b Department of Radiology, Chungbuk National University College of Medicine, Cheongju, Korea.

**Keywords:** acute respiratory distress syndrome, corticosteroid, cryptic miliary tuberculosis, serial radiologic study

## Abstract

**Rationale:**

Steroid is known to cause generalized immunosuppression, thereby increasing the risk of new infection or recurrence of tuberculosis. However, corticosteroid as a culprit for exacerbation of miliary tuberculosis—from a cryptic to an overt form—has rarely been described in the literature. Moreover, miliary tuberculosis is hardly diagnosed in a living patient as a primary cause of ARDS even in TB-endemic regions. To the best of our knowledge, this is the first case of a steroid-induced progression of cryptic miliary tuberculosis to ARDS, provided with clear depiction of its radiologic evolution.

**Patient concerns:**

A 36-year-old male was treated with corticosteroid under suspicion of adult onset still's disease for six-week history of fever. Within 2 weeks since the initiation of corticosteroid therapy, the patient experienced acute exacerbation of cryptic miliary tuberculosis, which evolved to an overt form, appearing as miliary nodules on both chest radiograph and HRCT. Then, his condition suddenly deteriorated to severe acute respiratory distress syndrome in less than a day.

**Diagnosis:**

The final diagnosis was miliary tuberculosis complicated by severe acute respiratory distress syndrome.

**Interventions:**

The patient was placed on classic quadruple anti-TB treatment (isoniazide, ethambutol, rifampin, and pyrazinamide).

**Outcomes:**

His fever subsided in about 6 weeks and 3 consecutive sputum AFB smears collected on different days were confirmed negative. Diffuse infiltrates on his chest x-ray were completely resolved.

**Lessons:**

The case described here draws a clinical and radiological picture of how an occult form of miliary TB evolved to an overt form with use of steroid, and then suddenly progressed to acute respiratory distress syndrome in an immunocompetent young male. This raises awareness on the potential risk of using corticosteroid in patients with cryptic miliary TB. There is formidable challenge in the diagnosis of miliary TB, especially in the early stages. Atypical or even normal outcomes of clinical, microbiochemical, and radiologic evaluation should not be overlooked and dedicated diagnostic work-up should be performed. For equivocal cases, active surveillance with serial radiographs can be helpful.

## 1 Introduction

Miliary tuberculosis (TB) is a fatal form of tuberculosis caused by massive lymphohematogenous dissemination of *Mycobacterium tuberculosis.*^[1]^ It can either occur as a primary progressive infection with an acute onset or insidiously develop from dormant bacilli (post-primary) with a subacute or chronic onset.^[2]^ In the past, miliary TB was thought to predominantly involve infants and children. Currently, it is increasingly being reported in adults, owing to the widespread use of immunosuppressive drugs and global HIV/AIDS pandemicity.^[3]^ The mortality rate related to miliary TB is about 25% to 30% in adults,^[4]^ and the rate increases to about 30% to 90% once it is complicated by acute respiratory distress syndrome (ARDS).^[5]^

Unfortunately, the diagnosis of miliary TB remains elusive even in TB-endemic areas, including South Korea, owing to atypical clinical presentations. In particular, the cryptic subtype of miliary TB, which lacks radiologic and other usual diagnostic characteristics, is frequently missed,^[6,7]^ resulting in irreversible impairment or demise of the patient.

Herein, we report the corticosteroid-induced exacerbation of cryptic miliary tuberculosis in a young male. He was placed on medium dose (12–20 mg/day) of methylprednisolone to treat adult onset still's disease (AOSD), which was supposedly responsible for six-week history of fever. His baseline chest radiograph and CT scans were normal. Two weeks after steroid therapy, he presented with dyspnea and chest radiography revealed diffuse nodular infiltrates in both lungs. Within a day, the patient rapidly deteriorated to ARDS requiring ICU management.

Following this case, we also provide a brief overview of the diagnostic challenges of miliary TB and shed light on how corticosteroids may have induced the exacerbation of occult miliary tuberculosis, ultimately resulting in ARDS.

## 2 Case presentation

This study was approved by the Institutional Review Board of Chungbuk National University Hospital (CBNUH 2020-09-008) and was CARE compliant. A 36-year-old man presented to the emergency department with a six-week history of fever. He described having short courses of cough, dyspnea, joint pain in both toes, and multiple pink colored rashes on the face and the upper body that wax and waned with fever. This patient had previously sought treatment from his primary physician and took antibiotics (which he did not recall the name of), but to no avail. His medical, social, exposure, and travel history was unremarkable, but was a smoker.

**Figure 1:**
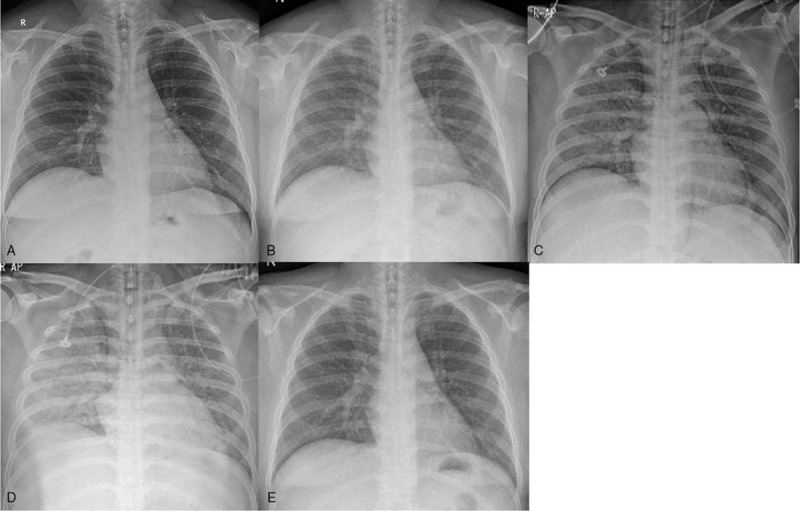
Radiological evolution of miliary tuberculosis with complicated acute respiratory distress syndrome in a 36-year-old male under corticosteroid for treatment of adult onset still's disease. **(**A) On the day of his visit to the emergency department, postero-anterior chest radiograph did not show any pathological findings. (B) Two weeks after initiation of corticosteroid therapy, subtle nodular opacities appeared in both lung fields on postero-anterior chest radiograph. Over the next 2 to 3 days, a rapid deterioration of clinical and radiological conditions ensued with (C) aggravation of diffuse nodular infiltrates and increased opacity in both lung fields, which (D) further progressed to diffuse alveolar involvement, showing “white lung” appearance. (E) Upon discharge, chest radiography showed complete resolution of the pathologic findings.

**Figure 2:**
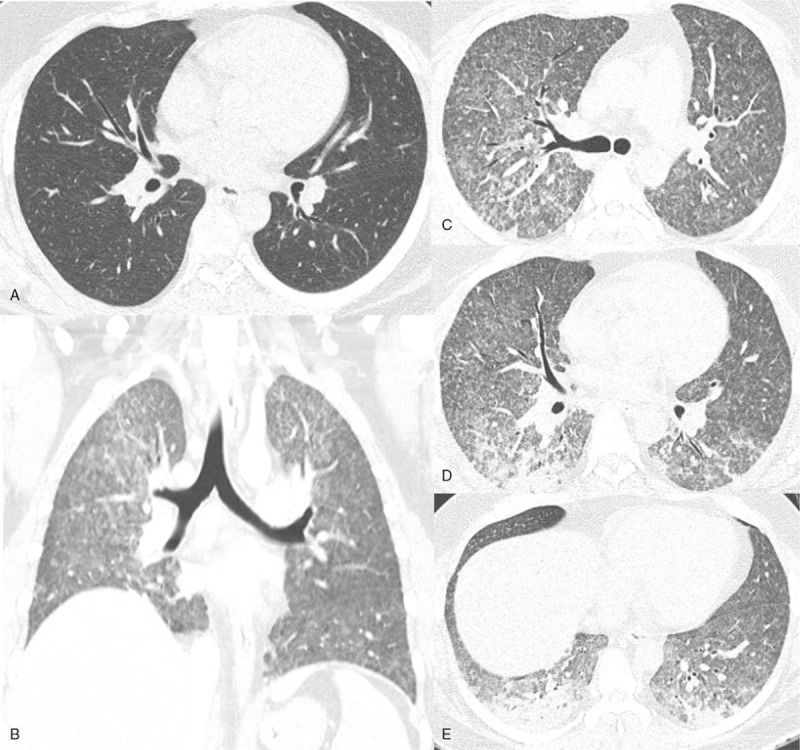
(A) Chest HRCT scanned initially upon his visit to the emergency department, without any pathological findings. (B-E) Chest HRCT scanned 2 weeks after initiation of corticosteroid therapy, when a rapid deterioration of clinical and radiological conditions occurred. (B, C, and D) Axial and (E) coronal images of HRCT show diffuse distribution of indistinct micronodules in all lobes of the lung. Notice there is no subpleural sparing. Also, there is non-homogeneous distribution of ground-glass opacities and dense consolidation in dependent regions, typically featured in ARDS.

Extensive biochemical and radiologic evaluations were conducted to uncover the origin of prolonged fever. The interferon-gamma releasing assay (IGRA) was positive (29.28), while there were no pathologic findings on chest radiograph (Fig. 1A) and high-resolution computed tomography (HRCT) (Fig. 2A), indicating latent TB or possibly cryptic miliary TB. The sputum acid-fast bacilli smear, *Mycobacterium tuberculosis* polymerase chain reaction (TB-PCR), and microbial culture were requested. There were elevated levels of serum ferritin (5634.9ug/L), lactate dehydrogenase (1411U/L), creatine phosphokinase (414U/L), and erythryocyte sedimentation rate (68 mm/hours). Rheumatoid factor and antinuclear antibodies were positive. The initial work-up diagnosis was possible AOSD with latent tuberculosis infection. Our rheumatology department expressed considerable doubt as to whether his symptoms and laboratory data satisfied the diagnostic criteria for AOSD, but he was empirically started on methylprednisolone (12 mg/day for 3 days and 20 mg/day for the next 11 days).

Two weeks after the initiation of corticosteroid therapy, his follow-up chest radiograph (Fig. 1B) showed subtle nodular opacities in both lungs. Two days later, he experienced exacerbation of dyspnea. A chest radiograph (Fig. 1C) and HRCT (Fig. 2B-2E) revealed diffuse nodular infiltrates in both lungs. Meanwhile, the sputum acid-fast bacilli (AFB) culture that was requested 3 weeks ago confirmed positive results for Mycobacterium tuberculosis. He was instructed to withhold steroids and was immediately admitted to the hospital for treatment of both radiologically and microbiologically confirmed miliary tuberculosis. He was started on classic quadruple anti-TB treatment (isoniazide, ethambutol, rifampin, and pyrazinamide).

Within 24 hours of admission, he deteriorated with a respiratory rate of 35bpm, and a PO2 of 78% on a non-rebreather mask at 15L/minutes of oxygen. He was intubated, transferred to the intensive care unit, and placed on mechanical ventilation. His PaO2/FiO2 ratio was about 94 mm Hg with PEEP 8 cm H_2_O, indicating severe ARDS.^[8]^ Chest radiograph (Fig. 1D) progressed to diffuse alveolar involvement, revealing a “white lung”. His condition further declined, requiring continuous renal replacement therapy (CRRT) for sepsis-induced acute kidney injury. Fortunately, his fever subsided in about 6 weeks, and 3 consecutive sputum AFB smears collected on different days were confirmed as negative. He was discharged home after ventilator weaning and discontinuation of CRRT. Diffuse infiltrates on his chest radiograph were completely resolved (Fig. 1E) upon discharge.

## 3 Discussion

Miliary tuberculosis is diagnosed when chest radiography or HRCT reveals a wide dissemination of tiny discrete pulmonary opacities that are generally uniform in size (2 mm or less in diameter in 90% of cases),^[1]^ or when there is a histopathological confirmation of miliary tubercles in tissue specimens.^[3]^ Notice the “or” condition, which means a disseminated pattern on chest radiograph is not mandatory in making the diagnosis. In fact, approximately 50% of patients with a confirmed diagnosis had normal chest radiographs^[2]^, in which case the disease was classified as cryptic, as in our case.

Cryptic miliary tuberculosis is defined when the typical radiology and clinical features are absent, whereas it is termed overt when the typical miliary infiltrate is observed on simple radiographs.^[6,7]^ The lack of typical x-ray findings imposes a diagnostic challenge, which often leaves the patients undiagnosed until autopsy.^[6,7,9–11]^ Normal radiographic findings upon admission cannot rule out the diagnosis and sequential chest radiographs or CT scans should be performed at least 2.5 weeks after the initiation of fever.^[3]^ Our patient underwent several chest radiographs or CT scans for follow-up observation, which facilitated timely diagnosis and intervention.

Adding perplexity to the problem, there is no gold standard for the diagnosis of miliary TB.^[12]^ Noninvasive rapid diagnostic tests for disseminated TB, including the sputum AFB smear, TB-PCR, and tuberculin skin test have yielded low sensitivities (61%, 79%, and 61%, respectively), whereas IGRA has reported a promising sensitivity of 90% and may be a beneficial adjunct study.^[13]^ In our case, AFB smear and TB-PCR of sputum and bronchial washing showed negative results, but IGRA was positive from the beginning. However, it was merely considered as latent TB, which could have been a misdiagnosis of cryptic miliary TB. In the end, mycobacterial culture confirmed the diagnosis, which took 3 weeks to grow. Therefore, it is of utmost importance to suspect the disease and perform a focused diagnostic work-up, or at least conduct serial surveillance chest radiographs.

The most crucial point in our case is how the patient progressed from a cryptic to an overt form of miliary TB. In retrospect, we speculate that recent steroid use^[14]^ was the culprit for the mayhem in our previously healthy patient. Steroids are a well-established predisposing factor of miliary TB along with childhood infections, HIV/AIDS, alcoholism, chronic kidney disease, connective tissue disorders, and underlying malignancy^[1]^. They hinder T-cell immunity, which is critical in the containment of *Mycobacterium tuberculosis*.^[15,16]^ It is likely that corticosteroid had created a quasi-immunocompromised state in our patient, leading to an overwhelming proliferation of the bacilli.^[4]^ Isoniazid prophylaxis prior to steroid therapy in patients with positive IGRA results is not routinely recommended, unlike other immunomodulating agents, such as infliximab and adalimumab.^[17,18]^

The development of ARDS in miliary TB is rare. According to previous reports, ARDS presents in 16% to 24% of the miliary TB cases,^[5]^ while M. tuberculosis accounted for only 3.6% of ARDS in a cohort from endemic regions.^[19]^ The pathogenesis of ARDS related to tuberculosis has yet been elucidated. One of the hypotheses postulated is massive spread of bacilli to the pulmonary circulation, resulting in inflammation, obliterative endarteritis, and alveolocapillary membrane damage,^[14]^ which is reminiscent of the pathogenesis of miliary TB. Furthermore, ARDS in miliary TB with regard to steroid use is even rarer. There have been randomized trials on how high-dose steroid therapy can increase the incidence of ARDS in general,^[20]^ but no systematically reviewed study pertaining to miliary TB was conducted. There was a case report on miliary TB complicated by ARDS in a patient who had been under high dose (40 mg/day) of corticosteroid for a year to treat rheumatoid arthritis,^[21]^ but our patient was given only medium dosage (12–20 mg/day)^[22]^ of methylprednisolone for 2 weeks. Future studies are needed to verify the dose-response relationship between corticosteroid use and complications of miliary TB.

## 4 Conclusion

The case described here draws a clinical and radiological picture of how an occult form of miliary TB evolved to an overt form with the use of steroids, and then suddenly progressed to acute respiratory distress syndrome in an immunocompetent young male. This raises awareness of the potential risk of using corticosteroids in patients with cryptic miliary TB. There is a formidable challenge in the diagnosis of miliary TB, especially in the early stages. Atypical or even normal outcomes of clinical, microbiochemical, and radiologic evaluation should not be overlooked, and dedicated diagnostic work-up should be performed. For equivocal cases, active surveillance with serial radiographs can be helpful.

## Author contributions

**Conceptualization:** Jin Young Yoo.

**Data curation:** Minji Song.

**Formal analysis:** Minji Song.

**Investigation:** Minji Song.

**Methodology:** Jin Young Yoo.

**Project administration:** Jin Young Yoo.

**Resources:** Jin Young Yoo.

**Supervision:** Sung Jin Kim, Jin Young Yoo.

**Validation:** Sung Jin Kim, Jin Young Yoo.

**Visualization:** Minji Song.

**Writing – original draft:** Minji Song.

**Writing – review & editing:** Minji Song.

## References

[1] Sharma SK Mohan A Miliary Tuberculosis. Microbiol Spectr 2017;5:2. 10.1128/microbiolspec.tnmi7-0013-2016 PMC11687475 28281441

[2] Abi-Fadel F Gupta K Acute respiratory distress syndrome with miliary tuberculosis: a fatal combination. J Thorac Dis 2013;5:E1–4. 23372959 10.3978/j.issn.2072-1439.2012.07.02 PMC3547991

[3] Mert A Arslan F Kuyucu T Miliary tuberculosis: epidemiologicaland clinical analysis of large-case series from moderate to low tuberculosis endemic country. Medicine (Baltimore) 2017;96:e5875. 28151863 10.1097/MD.0000000000005875 PMC5293426

[4] Sharma SK Mohan A Sharma A Challenges in the diagnosis & treatment of miliary tuberculosis. Indian J Med Res 2012;135:703–30. 22771605 PMC3401706

[5] Wakamatsu K Nagata N Kumazoe H Prognostic factors in patients with miliary tuberculosis. J Clin Tuberc Other Mycobact Dis 2018;12:66–72. 31720401 10.1016/j.jctube.2018.07.001 PMC6830168

[6] Deepika Patel RD Cryptic tuberculosis: a missed diagnosis and an unusual presentation. Int J Res Med Sci 2016;4:5463–5.

[7] Ozaras R Vatankulu B Mete B Cryptic miliary tuberculosis. QJM 2016;109:689–90. 27435669 10.1093/qjmed/hcw111

[8] Fanelli V Vlachou A Ghannadian S Acute respiratory distress syndrome: new definition, current and future therapeutic options. J Thorac Dis 2013;5:326–34. 23825769 10.3978/j.issn.2072-1439.2013.04.05 PMC3698298

[9] Bourbonnais JM Sirithanakul K Guzman JA Fulminant miliary tuberculosis with adult respiratory distress syndrome undiagnosed until autopsy: a report of 2 cases and review of the literature. J Intensive Care Med 2005;20:354–9. 16280410 10.1177/0885066605279150

[10] D’Odemont JP Montiel V Philippart I About an unusual systemic disorder: report of a cryptic miliary tuberculosis. Acta Clin Belg 2005;60:36–40. 15981704 10.1179/acb.2005.009

[11] Man MA Arghir OC Man S Fatal paradoxical cryptic miliary tuberculosis and immune reconstitution disease in a young non-HIV immunocompromised male patient: case report with autopsy findings. Rom J Morphol Embryol 2014;55:453–7. 24970001

[12] Sharma SK Mohan A Sharma A Miliary tuberculosis: a new look at an old foe. J Clin Tuberc Other Mycobact Dis 2016;3:13–27. 31723681 10.1016/j.jctube.2016.03.003 PMC6850233

[13] Yu SN Jung J Kim YK Diagnostic usefulness of IFN-Gamma releasing assays compared with conventional tests in patients with disseminated tuberculosis. Medicine (Baltimore) 2015;94:e1094. 26181542 10.1097/MD.0000000000001094 PMC4617092

[14] Hameed Raina Ab Fayaz Ahmad Bhat AB Khalid Hamid Changal Pulmonary tuberculosis presenting with acute respiratory distress syndrome (ARDS): A case report and review of literature. Egyptian J Chest Dis Tuberculosis 2013;62:655–9.

[15] Libert C Dejager L How steroids steer T cells. Cell Rep 2014;7:938–9. 24856295 10.1016/j.celrep.2014.04.041

[16] Han Y Kim SJ Lee SH High blood neutrophil-lymphocyte ratio associated with poor outcomes in miliary tuberculosis. J Thorac Dis 2018;10:339–46. 29600065 10.21037/jtd.2017.12.65 PMC5863179

[17] Esmail H Wilkinson RJ Minimizing tuberculosis risk in patients receiving anti-TNF therapy. Ann Am Thorac Soc 2017;14:621–3. 28459619 10.1513/AnnalsATS.201701-055ED PMC5427745

[18] Goletti D Petrone L Ippolito G Preventive therapy for tuberculosis in rheumatological patients undergoing therapy with biological drugs. Expert Rev Anti Infect Ther 2018;16:501–12. 29848120 10.1080/14787210.2018.1483238

[19] Singh K Hyatali S Giddings S Miliary tuberculosis presenting with ARDS and shock: a case report and challenges in current management and diagnosis. Case Rep Crit Care 2017;2017:9287021. 29318053 10.1155/2017/9287021 PMC5727565

[20] Hough CL Steroids for acute respiratory distress syndrome. Clin Chest Med 2014;35:781–95. 25453425 10.1016/j.ccm.2014.08.014 PMC4297198

[21] Pavlatos S Psallas M Makrilakis K Acute respiratory distress syndrome due to miliary tuberculosis in a patient with rheumatoid arthritis under corticosteroid therapy. Eur J Intern Med 2004;15:62–4. 15066653 10.1016/j.ejim.2003.12.003

[22] Buttgereit F da Silva JA Boers M Standardised nomenclature for glucocorticoid dosages and glucocorticoid treatment regimens: current questions and tentative answers in rheumatology. Ann Rheum Dis 2002;61:718–22. 12117678 10.1136/ard.61.8.718 PMC1754188

